# Diabetic nephropathy with minimal change disease: a case report

**DOI:** 10.3389/fendo.2025.1623272

**Published:** 2025-07-18

**Authors:** Pengwei Miao, Xiuting Mo, Xingkun Zhang, Mianzhi Zhang

**Affiliations:** ^1^ Tianjin University of Traditional Chinese Medicine, Tianjin Academy of Traditional Chinese Medicine Affiliated Hospital, Tianjin, China; ^2^ First Teaching Hospital of Tianjin University of Traditional Chinese Medicine, Tianjiin, China; ^3^ Tianjin Academy of Traditional Chinese Medicine Affiliated Hospital, Tianjin, China

**Keywords:** diabetes mellitus, diabetic kidney disease, minimal change disease, diabetic kidney disease combined with MCD, case report

## Abstract

**Background:**

Type 2 diabetes mellitus (T2DM) has become the main cause of end stage renal disease (ESRD) in the past decade. Diabetic kidney disease (DKD) is one of the most serious complications of diabetes. In clinical practice, the diagnosis of DKD is based primarily on clinical criteria, that is, patients almost do not undergo renal biopsy, leading to many non-diabetic kidney diseases (NDKDs) being misdiagnosed as DKD, thus increasing the incidence rate of DKD. The incidence of NDKD is also higher in those with DM. To date, few cases of minimal change disease (MCD) among those with DKD have been reported. Here, we report a case of diabetic nephropathy with pathological diagnosis, which was considered to be complicated with MCD according to the medical history, and was completely relieved after glucocorticoid treatment.

**Case description:**

A 49-year-old male patient with a diabetes duration of 3 years was admitted to our hospital mainly because of “bilateral lower extremity edema for 1 month”. The clinical manifestations were nephrotic syndrome and diabetic nephropathy, which were confirmed by renal biopsy. According to the medical history, DKD with MCD was considered. The patient received glucocorticoid for 6 months and was completely relieved of proteinuria.

**Conclusion:**

Renal biopsy is helpful to differentiate diabetes with chronic kidney disease (CKD). DKD and DKD with MCD can be differentiated in the early stage of DKD but are difficult to differentiate in the late stage of DKD. In clinical practice, for such patients, we should also diagnose them carefully based on their medical history to reduce the misdiagnosis rate.

## Introduction

With the development of the global economy, type 2 diabetes mellitus (T2DM) has become the main cause of end stage renal disease (ESRD) in the past decade (1). Diabetic nephropathy (DN) is one of the most serious complications of diabetes, which is caused by the thickening of the renal microvascular basement membrane, which causes exudative, diffuse, and nodular glomerulosclerosis, or complete hyaline changes, and finally progresses to uremia. In clinical practice, patients with T2DM combined with chronic kidney disease (CKD) almost do not undergo renal biopsy, leading to many non-diabetic kidney diseases (NDKDs) combined with T2DM being misdiagnosed as DKD, thus increasing the incidence rate of DKD (2, 3). For those with NDKD, appropriate treatment may result in improved outcomes. Minimal change disease (MCD) is a major cause of idiopathic nephrotic syndrome (NS) in both children and adults. Typically, it presents with “explosive” onset of NS over a period of days, not weeks or months, and it is characterized pathologically by the condition that is almost normal via light microscopy and shows diffuse effacement of foot processes via electron microscopy. To date, there are few cases of DKD with MCD. Because DN is often associated with podocyte injury, it increases the difficulty of differential diagnosis. We present the clinical diagnosis, treatment process, and outcome of a male patient with DKD combined with MCD. In this patient, accurate diagnosis and treatment based on renal pathology helped to improve the prognosis.

## Case report

### Chief complaint

A 49-year-old man was admitted to our hospital on 6 March 2022. His main complaint was “bilateral lower extremity edema for 1 month”.

### Present and past medical history

One month ago, the patient had depression edema in both bilateral lower extremity; there was no obvious cause, no fever, no malaise, no skin rash, and no photosensitivity. Routine urine test results (2022-2-23) were as follows: urine protein 3+, blood (−), and glucose (GLU) 3 +. Fasting blood glucose was 8. 1 mmol/L (2022-3-2),. Routine urine test results (2022-3-2) were as follows: protein 3+, microalbumin/creatinine (2022-3-2) was 33.9 mg/mmol, glucose 2+, urine microalbumin (2022-3-2) was 150 mg/L, and anti-phospholipase A2 antibody (PLA2R) (2022-3-2) was negative. An elevated blood glucose for 3 years was found for the patient, and he had not taken any mediations. He has undergone physical examination in the past 2 years and no abnormalities were found in his urine routine test. The patient has no other medical history. He reported no history of smoking and drinking. He married at 28 years of age; he had one son; both spouse and son were healthy. His father had T2DM and his mother died of unknown cause.

### Physical examination at admission

T, 36.8 °C; P, 85/min; R, 19 times/min; BP, 122/86 mmHg. The patient was 170 cm tall and weighed 65 kg. His body mass index (BMI) was 22.49 kg/m^2^. He had a clear consciousness, and there was no jaundice on the skin and sclera. His heart rate was 85 beats per minute, rhythm was regular, and no pathological murmurs were heard. The bilateral breath sound was clear without dry and moist rales. He has a soft abdomen without tenderness or rebound pain, and the liver and spleen were impalpable. There was no percussion pain in both kidney areas. Moderate pit edema of both lower extremities was observed.

### Imaging studies

Ultrasonography results: normal size for both kidneys. Chest computed tomography (CT) results: No obvious abnormalities were observed. Electrocardiography (ECG) results: The heart rhythm was sinus rhythm, with a regular rhythm of 85 beats per minute and no obvious abnormalities were observed.

### Laboratory results

Routine blood test results (2022-3-6): white blood cell (WBC) 9.78×10^9^, red blood cell (RBC) 4.62×10^12^, platelet (PLT) 273×10^9^, and neutrophil (N) 65%. Routine urine test results: urine protein 4+, occult blood±, RBC 0.61/Hp, and glucose (GLU) 2+. Renal function test results: blood urea nitrogen (BUN) 6.63 mmol/L, creatinine (Cr) 64 μmol/L, and uric acid (UA) 336 μmol/L. Liver function test results: albumin (ALB) 18.8 g/L, lipid: total cholesterol (TCH) 10.29 mmol/L, and triglyceride (TG) 2.40 mmol/L. The levels of serum albumin, serum creatinine, and urine protein of this patient on different dates are shown in **Table 1**. Glycosylated hemoglobin (HBA1c) was 8.3%. Fasting blood glucose was 9.8 mmol/L. Postprandial blood glucose was 13.8 mmol/L. Four items of coagulation, three items of cardiac enzymes, eight items of infection, antineutrophil cytoplasmic antibody (ANCA), anti-phospholipase A2 antibody (PLA2R), antinuclear antibody profile, autoantibodies, blood and urine immunofixation electrophoresis, immunoglobulins, and complements were normal.

**Table 1:** The levels of serum albumin, serum creatinine, and urine protein on different dates.

Laboratory results	24 March 2022	24 April 2022	5 May 2022	26 July 2022
Albumin (g/L)	18.8	31.6	36.8	41.2
Creatinine (mmol/L)	56	54	49	54
Urine protein (g/24 h)	12.803	0.798	0.073	0.132

The data in the second column are before treatment; the data in the next three columns are after treatment.

### Diagnosis

(1) NS and (2) T2DM.

#### Work-up

We gave the following treatment: (1) Monitoring of BP (telmisartan) and BG (liraglutide, dapagliflozin, and metformin), with a low-salt, low-fat diabetic diet; and (2) proposed the use of renal biopsy for definitive diagnosis. He has diabetes retinopathy (ou) and ametropia (ou) according to the consultation with an ophthalmologist.

**Figure 1:**
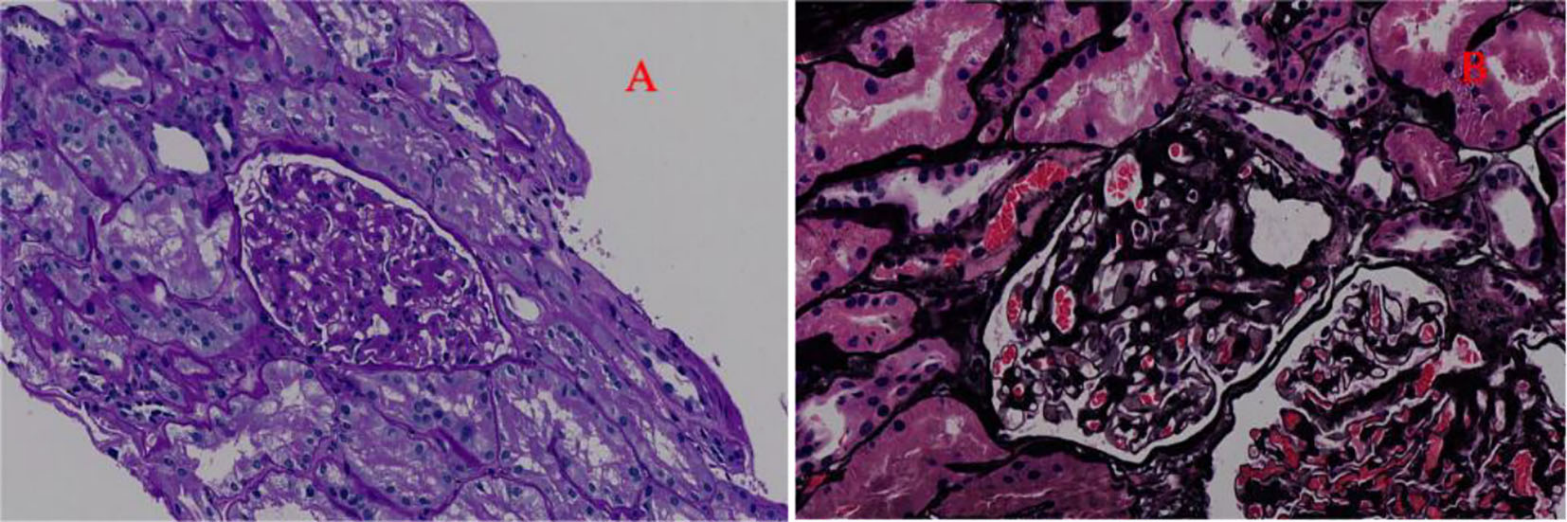
The optical microscope results of the patient. **(A)** Periodic acid–Schiff staining (200× magnification). This picture indicates mild to moderate to diffuse proliferation of mesangial cells and stroma. **(B)** Periodic acid–silver methenamine staining and Masson’s staining (200× magnification). This picture indicates moderate diffuse proliferation of mesangial cells and stroma.

After evaluation, the patient had no contraindications and underwent renal biopsy on 25 March 2022. Pathological results of renal biopsy: Immunofluorescence (IF): IgG− IgA− IgM+ C3− C1q− Fib− ALB− kappa− lambda−. Light microscopy showed mild to moderate diffuse proliferation of mesangial cells and stroma. The periodic acid–Schiff staining showed mild to moderate proliferation of mesangial cells and stroma (**Figure 1**). The periodic acid–silver methenamine staining and Masson’s staining showed moderate proliferation of mesangial cells and stroma (**Figure 1**). There were vacuoles and granular degeneration in renal tubular epithelial cells. Focal atrophy and a few protein casts can be observed in the lumen. Focal lymphocyte and monocyte infiltration with fibrosis, wall thickening of small arteries, intimal fibrosis and sclerosis, and lumen stenosis were also observed in the renal interstitium.

Mild to moderate proliferation of glomerular mesangial cells, homogeneous thickening of the glomerular basement membrane (GBM), occasional small deposits of electron-dense material in the mesangial region, and diffuse effacement of epithelial foot processes were observed upon electron microscopy examination. Small focal lymphatic and monocyte infiltration was observed in the renal interstitium. Thickening of the walls, visible hyaline degeneration, and narrowing of the lumen were observed in the small arteries.

**Figure 2:**
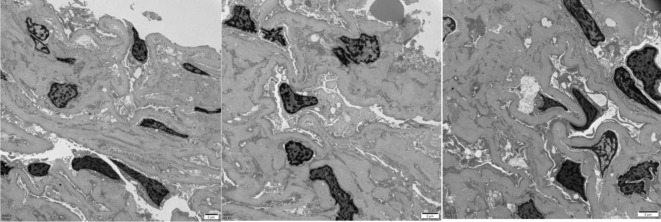
The electron microscope results of the patient.

These results were consistent with mild to moderate mesangial proliferative DN (IIa) (**Figure 2**). However, diffuse effacement of epithelial foot processes was observed by the electron microscopy examination, and we consider that the patient also had MCD based on the patient’s medical history. Glucocorticoid (methylprednisolone: 32 mg, q.d.) therapy was conducted for 6 months, after which NS was completely relieved. Urine routine test on 11 October 2022: urine protein −, occult blood −, glucose (GLU) 2+.

## Discussion

We here report one case of a concomitant occurrence of MCD with DKD. The patient had complete and sustained remission after steroid treatment. The kidney biopsy indicated DKD and clinical outcome was consistent with MCD. This case emphasizes the essential role of renal biopsy and medical history in patients with diabet es who present recent development of nephrotic proteinuria.

In clinical practice, patients with T2DM combined with CKD almost do not undergo renal biopsy (4), leading to many NDKD combined with T2DM being misdiagnosed as DKD, thus increasing the incidence rate of DKD (2, 3). According to pathological analysis of CKD combined with DM patients, they can be divided into DKD, NDKD, and NDKD with DKD. These three entities can be reliably distinguished only by kidney biopsy (3). The incidence rate of NDKD combined with DKD in patients with T2DM is 33% –72.5%. Glomerulonephritis and FSGS are the most common pathologies (4). NDKD includes glomerulonephritis, MCD, primary and secondary focal segmental sclerotic nephropathy (FSGS), and paraprotein-related kidney damage.

In most cases of MCD in patients with diabet es, renal biopsy shows either no significant histologic alterations or only mild mesangial sclerosis and glomerular basement membrane thickening, consistent with DN (5, 6). In all of these cases, foot process effacement was usually described as extensive. In some cases, the diagnosis of MCD in the setting of nodular diabetic glomerulosclerosis is problematic as diffuse foot process effacement and nephrotic proteinuria are common to both of these conditions. Diffuse and extensive are always subjective and indistinguishable. In fact, MCD should only be considered where there has been abrupt onset of NS and a documented absence of proteinuria in recent times, which shows the importance of medical history in differential diagnosis (7).

The patient had an unknown history of diabetes, diabetes retinopathy, and proteinuria. If renal biopsy was not performed, the patient can be clinically diagnosed with DKD, and the prognosis would be poor. After the renal biopsy was reported, the diagnosis of DKD became clearer, and owing to the medical history and diffuse effacement of podocyte processes, MCD was considered. He experienced complete remission of NS after treatment targeted MCD. It was reported that steroid hormone and immunosuppressive therapy might improve prognosis in at least 50% of patients with DM with CKD, and an accurate diagnosis based on kidney biopsy could improve the renal lesion (8, 9).

## Conclusion

Renal biopsy may help to differentiate diagnosis of DKD, NDKD, and NDKD combined with DKD. Clinicians should carefully identify patients with unknown DM duration combined with NS in order to reduce misdiagnosis and to improve prognosis.

## Data Availability

The original contributions presented in the study are included in the article/**Supplementary Material**. Further inquiries can be directed to the corresponding author.
