# Supplementary figures and images for: Diabetic nephropathy with minimal change disease: a case report
Source: Front Endocrinol (Lausanne). 2025 Jul 18;16:1623272. doi: 10.3389/fendo.2025.1623272 (PMC12313474; doi:10.3389/fendo.2025.1623272)

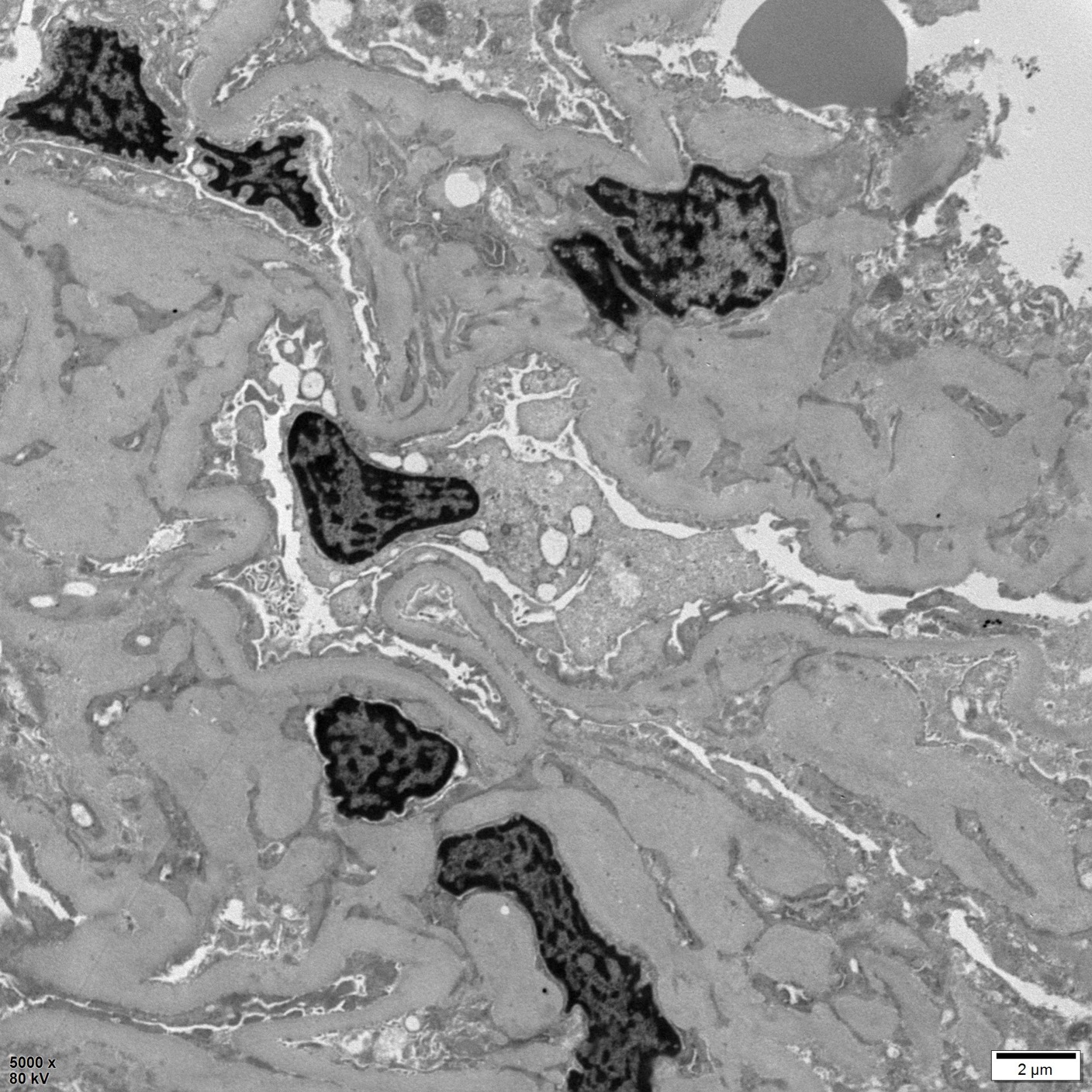

Supplement: Supplementary file 1 [file Image1.jpeg]

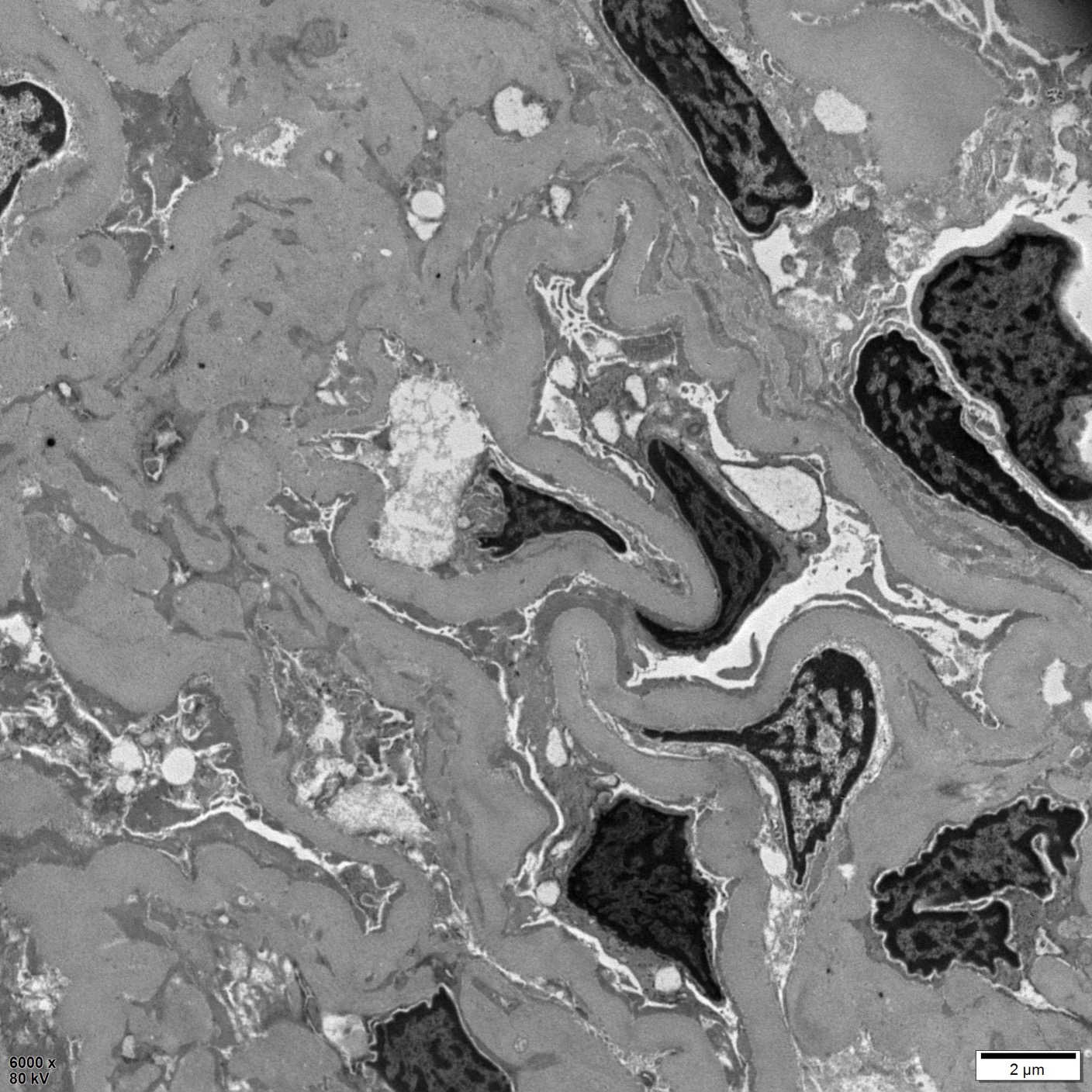

Supplement: Supplementary file 2 [file Image2.jpeg]
